# Comparative Analysis of Sustainable Extraction Methods and Green Solvents for Olive Leaf Extracts with Antioxidant and Antihyperglycemic Activities

**DOI:** 10.3390/antiox13121523

**Published:** 2024-12-12

**Authors:** Nils Leander Huamán-Castilla, Luis Omar Mamani Apaza, Franz Zirena Vilca, Erick Saldaña, Yemina Karen Diaz-Valencia, María Salomé Mariotti-Celis

**Affiliations:** 1 Escuela Profesional de Ingeniería Agroindustrial, Universidad Nacional de Moquegua, Prolongación Calle Ancash s/n, Moquegua 18001, Peru; mluis1707@gmail.com (L.O.M.A.); esaldanav@unam.edu.pe (E.S.); 2 Laboratorio de Tecnologías Sustentables para la Extracción de Compuestos de Alto Valor, Instituto de Investigación para el Desarrollo del Perú (IINDEP), Universidad Nacional de Moquegua, Moquegua 18001, Peru; 3 Laboratorio de Contaminantes Orgánicos y Ambiente, Instituto de Investigación para el Desarrollo del Perú (IINDEP), Universidad Nacional de Moquegua, Moquegua 18001, Peru; fzirenav@unam.edu.pe; 4 Departamento Académico de Ingeniería de Industrias Alimentarias, Universidad Nacional de San Agustín, Arequipa 04001, Peru; ydiazva@unsa.edu.pe; 5 Faculty of Medicine, Nutrition and Dietetics School, Universidad Finis Terrae, Pedro de Valdivia 1509, Providencia, Santiago 7501015, Chile

**Keywords:** antioxidant compounds, pressurized liquid extraction, ultrasound assistant extraction, glucosidase/amylase, diabetes mellitus type 2

## Abstract

Olive leaves are agro-industrial waste that pose an environmental management problem. However, they contain polyphenolic compounds with important bioactive properties beneficial to human. This study aimed to evaluate the effectiveness of two extraction technologies (pressurized liquid extraction and ultrasound-assisted extraction) combined with green solvents (pure water, 15% ethanol, and 15% glycerol) at 50 °C and 70 °C. The goal was to obtain extracts with both antioxidant capacity and antihyperglycemic properties from olive leaves. Pressurized liquid extraction with 15% glycerol at 70 °C was the most effective method for obtaining extracts rich in total polyphenols (19.46 mg GAE/g dw), with an antioxidant capacity of 4.11 mg/mL (inhibition capacity: IC50) and 500.26 µmol TE/g dw. For both extraction methods at 70 °C, glycerol was more effective at recovering phenolic acids, stilbenes and secoiridoid; while ethanol was more effective for recovering flavonols and phenylethanoids. Oleuropein was the most important polyphenol extracted from both pressurized liquid and ultrasound-assisted extractions, with concentrations of 171.48 µg/g dw and 246.70 µg/g dw, respectively. The extract obtained from pressurized liquid extraction with 15% ethanol at 70 °C exhibited significant inhibition (70%) of α-glucosidase enzymes, similar to the reference drug acarbose. In contrast, these extracts showed low inhibitory activity against the α-amylase enzyme. These findings can be applied to the development of functional foods and nutraceutical supplements aimed at managing postprandial glycemic response, offering a natural alternative for supporting type 2 diabetes management.

## 1. Introduction

Plant extracts have been widely used for centuries as natural medicine due to their positive effects on human health [1]. Extracts obtained from different parts of plants, such as leaves, roots, flowers, and fruits, contain high concentrations of polyphenols. These compounds have shown antioxidant, anti-inflammatory, and antimicrobial properties that have garnered interest from the industry [2]. For example, extracts from *Gymnema sylvestre* are recognized for their ability to reduce hyperglycemia [3] while extracts from *Azadirachta indica* and Aloe vera exhibit antiparasitic and anti-inflammatory activities, respectively [4,5]. This has driven ongoing research into sustainable, cost-effective natural extract sources with potential therapeutic and industrial applications.

The olive tree, known as *Olea europaea*, is a tree that thrives in Mediterranean climates whose fruit (olives), and its by-products (olive oil) are widely consumed in the global market [6,7]. Thus, 56 countries grew 1.5 billion olive trees, covering 11 million ha during 2023 [8]. In Peru, 28,000 ha of olive trees are used for producing olives and their derivatives like oil (virgin and extra virgin) where Tacna, Arequipa, and Moquegua produce 176,000, 41,000, and 684 tons of olives per year, respectively [9]. However, the process of harvesting and processing olives generates agro-industrial waste such as skins, seeds, and leaves which contribute to environmental pollution [10,11]. In particular, olive leaves are a rich natural source of polyphenols with several bioactive properties, including antioxidants, anti-inflammatory, antimicrobial, antiproliferative, antiarrhythmic, antihypertensive, hypoglycemic, and hypocholesterolemic effects [12,13,14].

In olive leaves, polyphenols are compounds metabolized as a defense mechanism against external factors, whose chemical structure presents phenolic rings and hydroxyl groups, and they can be categorized like phenolic acids, flavanols, flavonols, stilbenes, secoiridoids, and other polyphenolic compounds [15,16]. Polyphenols exhibit varying degrees of solubility in organic solvents due to hydrogen bonding and dipole–dipole interactions [17]. Conventional extraction methods like Soxhlet, maceration, and distillation have been developed for polyphenol extraction [15]. However, these methods have several disadvantages, such as prolonged processing times and the use of environmentally hazardous toxic solvents (acetone, hexane, methanol) [18,19,20].

Alternative technologies such as pressurized liquid extraction (PLE) and ultrasound-assisted extraction (UAE) using green solvents have gained importance due to their efficiency and sustainability [21,22,23]. PLE employs elevated pressures (10 atm) and temperatures (60–250 °C) which enhance solvent penetration and speed up the extraction process, resulting in high yields of polyphenols in short time (<15 min) [24]. In turn, UAE operates at lower temperatures (<90 °C) and shorter extraction times (<30 min), which disrupts cell structures increasing the mass transfer and therefore the extractability of polyphenols [25]. Both PLE and UAE offer versatile options for developing extracts with antioxidant and antihyperglycemic properties, which have potential applications in functional foods and nutraceuticals [26,27].

Different studies have been conducted using both technologies with eco-friendly protic solvents to extract polyphenols from plant matrices. In particular, glycerol and ethanol are protic solvents and can donate protons (H^+^) due to the presence of a hydrogen atom attached to an electronegative atom like oxygen, which can establish hydrogen bonds with functional groups of polyphenols [28,29,30]. Rivera-Tovar et al. [31] found that raising the temperature from 80 to 200 °C in PLE using water–ethanol mixtures with intermediate ethanol concentrations (5–80%) enhances the recovery of polyphenols from maqui leaves. Similarly, Huaman-Castilla et al. [32] demonstrated that the use of PLE at high temperatures (90–150 °C) with 50% water–ethanol mixtures boost the recovery of polyphenols from grape pomace.

Regarding UAE, Şahin and Şamli [33] demonstrated that this technology (50 Hz) combined with a 50% water–ethanol mixture at room temperature was 40% more effective in recovering polyphenols from olive leaves than using pure water. Additionally, Bin Mokaizh et al. [34] found that UAE (20 kHz) with a 40% water–ethanol mixture at room temperature increases polyphenol recovery from *C. gileadensis* leaves by 20% compared to a 20% water–ethanol mixture under the same conditions. While both methods demonstrate high yields in recovering polyphenols, a comparative study between PLE and UAE is essential to determine the most suitable technique for extracting these bioactive compounds. This study assessed two clean technologies—UAE and PLE—using protic solvents (pure water, ethanol, and glycerol) at moderate temperatures to recover polyphenols from olive leaves.

Several studies have demonstrated that extracts obtained from agro-industrial waste have the potential to reduce oxidative stress [35,36]. Polyphenols present in these extracts have demonstrated promising potential in inhibiting key enzymes involved in carbohydrate digestion, such as α-amylase and α-glucosidase. These enzymes play a crucial role in managing postprandial hyperglycemia in diabetes [37,38]. For example, polyphenol extracts have reported strong inhibitory effects on both α-amylase and α-glucosidase, comparable to the antidiabetic drug acarbose, indicating a promising potential for natural alternatives in diabetes management [39,40,41]. On the other hand, walnut green husk extracts demonstrated inhibition on α-glucosidase when ultrasound-assisted extraction was applied to water–ethanol mixture (45%) for 50 min at room temperature [42]. These findings suggest that agro-industrial waste can be effectively utilized to produce enzyme-inhibitory extracts. Thus, the goal was to identify the most effective extraction technique for this agro-industrial residue, as well as evaluate the inhibitory effects of these extracts on carbohydrate-digesting enzymes like α-amylase and α-glucosidase, which are key targets in managing postprandial hyperglycemia associated with type 2 diabetes.

## 2. Materials and Methods

### 2.1. Chemicals and Analytic Reagents

Solvents for extraction were obtained from Sigma Aldrich (St. Louis, MO, USA) such as glycerol (>99%), ethanol (>99%), and water (MS Grade). Similarly, specific polyphenols (standard) such as gallic acid (>99%) catechin (>98%), procyanidin A2 (>98%), epicatechin (>99%), kaempferol (>98%), resveratrol (>98%), quercetin (>99%), hydroxytyrosol (>99%), tyrosol (>99%), and oleuropein (>99%) were purchased from Sigma Aldrich Co., Ltd. (St. Louis, MO, USA).

### 2.2. Samples

A total of 10 kg of olive leaves was recollected in June 2023 (coordinates: 17°0′8.64′′ S; 72°5′48.05′′ W), taking into consideration factors such as the age and variety of the tree (10 years and Sevillian) as well as the quality of the leaves, which were elliptical, green, and free of any physical defects. The procurement process involved gathering the leaves during pruning after the harvest of the olive fruit. The supplier of these leaves was Agroindustrias Unidas del Perú S.A.C. (Fundo Olivers, Islay, Arequipa), a respected company in the industry. After being collected, the olive leaves were washed with distilled water to remove any surface contaminants. Then, the leaves were spread out in a single layer and air-dried at room temperature (approximately 25 °C) for 48 h to get rid of excess surface moisture. After, the leaves were further dried in a convection oven set at 35 °C for 24 h. This controlled drying process ensured that the leaves reached a consistent moisture content, which was determined on a dry basis. Then, the sample’s particle size was reduced (0.5 mm) using a pulse mill (Analysette 3 Pro, Fritsch, Idar-Oberstein, Germany). The resulting sample was stored in Ziploc bags at −20 °C until further use.

### 2.3. Ultrasound-Assisted Extraction (UAE) of Polyphenols

The conditions process for obtaining extracts from olive leaves were based on a previous study conducted by Giacometti et al. [43]. In brief, 10 g of dried olive leaves was placed in a beaker (250 mL). After, an ultrasound-assisted extraction system equipped with a probe tip (ø: 4 mm) (CY-500, Optic Ivymen Systems™, COMECTA^®^, Barcelona, Spain) was used to extract the compounds. The ultrasound system operated at 20 kHz with and output power of 70% for 30 min. The solvent composition used in this study was selected based on our previous research findings [26,44]. In this sense, solvents like ethanol (15%, *v*/*v*), glycerol (15%, *v*/*v*), and pure water were combined at moderate temperatures (50 and 70 °C). These conditions yielded a solid sample to extract a volume ratio of 1:10. The extracts obtained were centrifuged at 4000 RPM for 10 min using a centrifuge (Pro-analityt.C2004, Centurion Scientific Ltd., Bosham, UK). The supernatant was filtered using filter paper. Finally, the extracts were stored in amber vials at a temperature of −20 °C until further analysis.

### 2.4. Pressurized Liquid Extraction (PLE) of Polyphenols

To compare the efficiency in polyphenol recovery, extraction temperatures were set at 50 and 70 °C for both methods (PLE and UAE) while the fixed parameters used in this study were selected based on our previous research findings [26]. The methodology proposed by Huamán-Castilla et al. [24] was utilized with some modifications. In brief, a mixture of 10 g of olive leaves (dry weight) and quartz sand in a 1:4 *w*/*w* ratio was placed in a 100 mL extraction cell within a pressurized liquid system (ASE 150, Dionex, Thermofisher, San Jose, CA, USA). Extraction solvents such as pure water, ethanol (15%, *v*/*v*), and glycerol (15%, *v*/*v*) were employed at 50 °C and 70 °C. These settings resulted in a solid sample/extract volume ratio of 1:10. The crude extracts were preserved in amber vials at −20 °C until further analysis.

### 2.5. Total Polyphenol Content

To quantify the total polyphenol content present in the extracts obtained, the Folin–Ciocalteu method proposed by Singleton and Rossi [45] was employed with some modifications. In brief, a mixture of 0.5 mL of extract, 0.25 mL of Folin–Ciocalteu reagent (1N), and 3.75 mL of distilled water were prepared. Then, a 10% *w*/*v* sodium carbonate solution was added and allowed to react at room temperature in the dark for one hour. The solution was analyzed using a spectrophotometer (UV–Vis Genesys 150, Thermofisher, San Jose, CA, USA) at a wavelength of 765 nm. Previously, a calibration curve (R^2^ > 0.9988) was generated using different of gallic acid concentrations from 10 to 100 mg/L. The results of the experiment were expressed as milligram of gallic acid per gram of dry weight (mg GAE/g dw), which was calculated using the following equation:

$$IC=\left(1-\frac{A_{s}}{A_{c}}\right)\times 100$$

where IC is inhibitory concentration, A_s_ is the absorbance of samples, and A_c_ is the absorbance of control.

### 2.6. Antioxidant Capacity by DPPH (2,2-Difenil-1-Picrilhidracilo) Method

To determine the antioxidant capacity of olive leaf extracts, the DPPH radical inhibition method proposed by Brand-Williams et al. [46] was used. In brief, a solution of 0.1 mL of the extract with 3.9 mL of DPPH solution was prepared and then incubated for 30 min in darkness. The reduction in the DPPH radical was measured with a UV–Vis spectrophotometer (UV–Vis Genesys 150, Thermofisher, San Jose, CA, USA). Simultaneously, methanol and methanolic DPPH solutions were used as positive and negative controls, respectively. The results were expressed as IC50 values (mg/mL), which indicates the extract concentration necessary to inhibit 50% of the DPPH radical activity.

### 2.7. Antioxidant Capacity by Oxygen Radical Absorbance Capacity (ORAC) Method

The extracts were analyzed using ORAC with the assistance of a microplate reader (Synergy/HTX, Biotek Instruments Inc., Winooski, VT, USA), following the methodology proposed by Chirinos et al. [46]. In brief, a 75 mM PBS buffer solution was prepared with type I water (pH: 7.4). Then, fluorescein (55 nM), AAPH (153 mM), and Trolox as a reference standard (8, 16, 24, 32, and 40 µM) were diluted with the PBS buffer, and each sample was likewise diluted. After, 25 µL of each sample, Trolox, and a blank (PBS buffer) were added to a 96-well black microplate, which was inserted into the microplate reader. The reader automatically added 250 µL of fluorescein into each well, and the mixture was incubated at 37 °C for 10 min. Then, 25 µL of AAPH was injected into each well. Fluorescence readings were taken every minute for 50 min at 485 (excitation) and 520 nm (emission). Finally, the ORAC values were calculated using the area under the curve and expressed as µmol TE/g dw (micromoles of Trolox equivalent per gram of dry weight).

### 2.8. α-Amylase Activity

The method for measuring the inhibition of α-amylase activity was adapted from Huaman-Castilla et al. [47]. Initially, each extract was dried using nitrogen gas, redissolved in DMSO (dimethyl sulfoxide), and then filtered to prepare a 10 mg/mL sample stock solution. The experimental dilutions ranged from 0.1 to 3000 µg/mL in phosphate buffer (pH 6.9). For the assay, 100 µL of each dilution was mixed with 100 µL of 1% starch solution in a 20 mm sodium phosphate buffer (pH 6.9) and incubated at 25 °C for 10 min. After, 100 µL of porcine pancreatic α-amylase solution (0.5 mg/mL) was added to each sample, and incubation continued for an additional 10 min at 25 °C. The reaction was terminated by the addition of 200 µL dinitro salicylic acid reagent, and the mixture was heated at 100 °C for 5 min. Afterward, 50 µL from each sample was transferred to a 96-well microplate, diluted with 200 µL of water per well, and the absorbance was measured at 540 nm to determine the enzymatic activity.

$$Amylase\;Activity=\frac{Absorbance\;of\;extract}{Absorvance\;of\;control}\times 100$$

The control is the enzyme–substrate reaction in the absence of inhibitors. The effect of the pharmacological inhibitor, acarbose, was also determined following the same protocol as previously described.

### 2.9. α-Glucosidase Activity

The ability of each extract to inhibit α-glucosidase activity was measured using the method described by Huaman-Castilla et al. [47]. The samples were prepared in the same way as for the α-amylase activity assay. The inhibitory effect of each extract was measured at concentrations from 0.1 to 1000 μg of Carménère pomace /mL in 100 mM sodium phosphate buffer (pH 6.9). A volume of 50 μL of extract solution and 50 μL of 5 mM p-nitrophenyl-α-D-glucopyranoside (PNPG) solution (in phosphate buffer) was mixed in a 96-well microplate and incubated at 37 °C for 5 min. Then, phosphate buffer (100 μL) containing 0.1 U/mL of μ-glucosidase (from *S. cerevisiae*) was added to each well. The absorbance at 405 nm was recorded for 15 min using a microplate reader at 37 °C. The effect of the commercial inhibitor on α-glucoside activity was also determined, and data were processed as in the previous assay.

### 2.10. Polyphenol Profile

Specific polyphenols were quantified using the methodology proposed by Huaman Castilla et al. [44] with some modifications. Previously, solid-phase extraction (SiliaPrep C18 6 mL, 500 mg SPE cartridges) for sample preparation was used. Polyphenol standards were diluted and injected into an ultra-high-performance liquid chromatograph (Agilent 1290 Infinity II, Santa Clara, CA, USA) coupled with a diode array detector and a reversed-phase column (Poroshell EC-C18, 2.1 mm × 150 mm × 1.9 μm). Then, pure water and acetonitrile with 0.1% formic acid were used as mobile phase at 30 °C. The gradient at 0.3 mL/min consisted of three steps: for 2 min, the mixture was 95% A and 5% B; after, a mixture with 60% A and 40% B was used for 15 min; and 95% A and 5% B were used for 18 min. Finally, the retention time and area under the curve of each of the analyzed standards were compared to quantify the specific polyphenols (Table 1). The results were expressed in micrograms of specific polyphenols per gram of dry weight and the analysis was performed in triplicate.

### 2.11. Statistical Analysis

Two extraction technologies (ELP and EAU) were studied using a 3×2 full factorial design with three repetitions. This design allowed for the evaluation of the effects of different solvents (pure water, 15% *v*/*v* ethanol, and 15% *v*/*v* glycerol) and moderate temperatures (50 °C and 70 °C) on response variables, including total polyphenol content, antioxidant capacity (DPPH and ORAC), polyphenol profile, and enzymatic activity. The data were analyzed using Shapiro–Wilk tests to verify the normality of residuals and Levene’s test to check homogeneity of variance. Then, a two-way analysis of variance (ANOVA) was used to examine the main effects and interactions between factors (*p* value < 0.05). Following the ANOVA, Tukey’s test was applied for post hoc multiple comparisons to identify significant differences between groups. For each comparison, we reported *p*-values, and confidence intervals were set at 95% to ensure robust interpretation of the results.

Principal Component Analysis (PCA) was conducted to assess how different combinations of extraction methods (pressurized liquids and ultrasound), temperatures (50 °C and 70 °C), and solvent types (water, ethanol, and glycerol) affect the polyphenol profile. The analysis utilized the Pearson correlation matrix, treating the experimental combinations as individuals and the polyphenol compounds as active variables, while their respective families were considered supplementary variables. The analyses were performed using R software version 4.3.2 (R core Team, 2024), specifically leveraging the FactoMineR package to identify meaningful patterns and associations within the dataset.

## 3. Results and Discussions

### 3.1. Total Polyphenol Content

**Figure 1:**
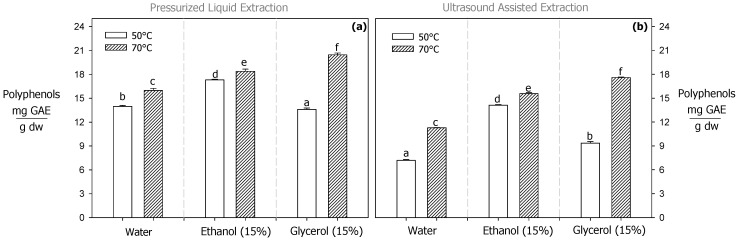
Total polyphenol content in extracts obtained by PLE (**a**) and UAE (**b**). Polyphenol content is expressed as mg of gallic acid equivalent (GAE) per gram dry weight (g dw). Different letters indicate significant differences (*p* < 0.05) between the extraction solvents for each extraction process.

For the PLE process, increasing the temperature from 50 to 70 °C improved the total polyphenol content by 14%, 8%, and 50% with pure water, 15% ethanol, and 15% glycerol, respectively (Figure 1a). Some studies have reported that water–glycerol mixtures significantly enhance polyphenol extraction compared to water–ethanol mixtures and pure water under atmospheric conditions, suggesting a superior efficacy of glycerol in the extraction process. Apostolakis et al. [48] found that a 9.3% water–glycerol mixture at 80 °C recovered 9.4% more polyphenol from olive leaves compared to a 60% water–ethanol mixture under the same conditions. Similar behavior was reported by Karakashov et al. [49], who observed thar the use of a 10% water–glycerol mixture at 70 °C improved the extraction of phenolic compounds from *Hypericum perforatum* by 13.8% compared to pure water under the same conditions. Under subcritical conditions, Huamán-Castilla et al. [49] concluded that a 30% water–glycerol mixture recovered 26% more polyphenols from grape pomace than a 30% water–ethanol mixture at 150 °C. Glycerol has greater capacity to form hydrogen bonding (α:1.21) compared to other solvents like water (α:1.07) and ethanol (α:0.98) [50]. In addition, during the PLE process, an increase in temperature reduces polarity and viscosity, improving the extraction of polyphenols [32,51].

For the UAE process (70 °C), the use of 15% water–glycerol mixture recovered 56% and 13% more polyphenols compared to the 15% water–ethanol mixture and pure water, respectively (Figure 1b). A similar trend was described by Sucharitha et al. [52] who found that using water–glycerol mixture (3:1) at 60 °C during UAE recovered 68% and 34% more polyphenols from olive leaves compared to a water–ethanol mixture (3:1) and pure water, respectively, under the same conditions. Temperatures above 50 °C enhance the cavitation phenomenon in low-vapor-pressure solvents like glycerol; consequently, a greater polyphenol content is released from the plant raw material [53]. Both extraction methods (PLE and UAE) were able to recover substantial concentrations of total polyphenols. However, the PLE process with 15% water–glycerol mixture at 70 °C recovered 11% more polyphenol than the UAE process at the same conditions (Figure 1a,b). Although the UAE process utilizes ultrasonic waves to induce cavitation and disrupt the plant matrix, its ability to enhance solvent penetration is limited compared to the high pressure and temperature used in PLE. In addition, the elevated pressure in PLE can change the solvent’s properties, enabling the dissolution of a broader range of polyphenolic compounds [21,47]. Meanwhile, UAE operates at atmospheric pressure, which constrains the solvent’s properties and reduces the solubility of certain polyphenols.

### 3.2. Antioxidant Capacity

**Figure 2:**
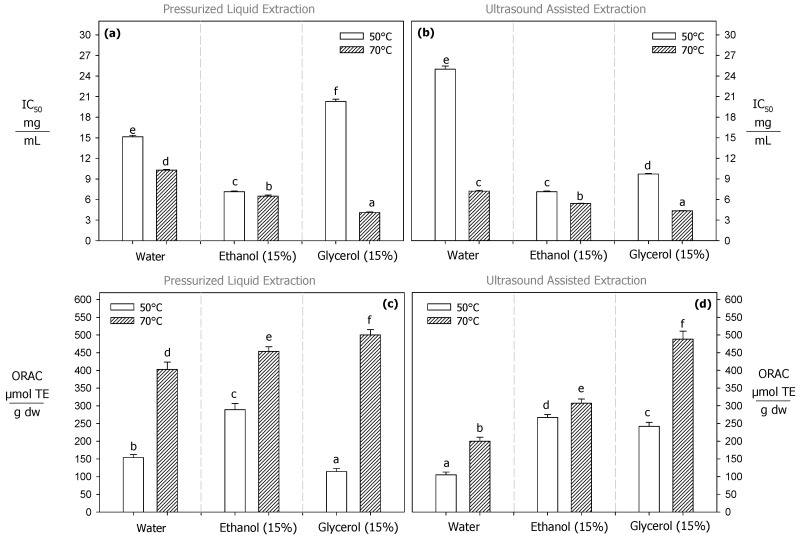
Antioxidant capacity of extracts obtained through PLE and UAE processes. IC_50_: radical inhibition capacity is expressed as mg of extract per mL of solution. Note: (**a**,**b**) represents the IC50 values for ELP and UAE, respectively; (**c**,**d**) represents the ORAC values for ELP and UAE, respectively. ORAC is expressed as micromoles of Trolox equivalent per gram of dry weight (g dw). Different letters indicate significant differences (*p* < 0.05) between the extraction solvents for each extraction process.

Polyphenols present hydroxyl (OH) groups and can transfer hydrogen atoms to reduce the DPPH radical [54]. In this sense, a high potential of radical inhibition in an extract is related with a high polyphenol content. For both PLE and UAE, the IC_50_ values decreased with increasing temperature. Under subcritical and ultrasound-assisted conditions, the amount of extract necessary to decrease DPPH radical activity by 50% (IC_50_) was 10.2, 6.5, and 4.1 mg/mL (Figure 2a) for pure water, 15% water–ethanol, and 15% water–glycerol mixtures, respectively, and 7.2, 5.4, and 4.7 mg/mL (Figure 2b) for the same mixtures during the PLE process, respectively. Interestingly, the optimal condition to inhibit 50% of DPPH radical activity was the 15% water–glycerol mixture at 70 °C for both PLE and UAE, with no significant differences observed (*p* > 0.05) (Figure 2a,b). A similar behavior was reported by Huaman et al. [47] who found that an increase from 90 to 150 °C reduced by 66% and 53% the IC_50_ value with ethanol (15%) and glycerol (15%), respectively. Mamani-Pari et al. reported [26] that an increase from 50 to 70 °C reduced by 72% and 33% the IC_50_ value with pure water and ethanol (30%), respectively.

Another method for evaluating the antioxidant capacity of an extract is the ORAC method, which quantifies the ability of polyphenols to scavenge oxygen radicals generated in the test system. This mechanism closely resembles the interactions between antioxidants and radicals in vivo, providing a more accurate simulation of antioxidant activity in the human body. Consequently, the ORAC method serves as a more reliable indicator of bioactivity compared to the DPPH assay [55].

In this study, similar to the DPPH evaluation, both processes (PLE and UAE) at higher temperatures (70 °C) enhanced the antioxidant capacity of the extracts, as determined by the ORAC method (Figure 2c,d). The increase in temperature is expected to improve the mass transfer of polyphenols and consequently enhance the antioxidant capacity of the extracts [56]. In this sense, in previous studies on polyphenol PLE from natural sources, we found that the polarity and hydrogen-bonding capacity of the solvent play a crucial role in determining the antioxidant properties of the extract [24,44]. Water–glycerol mixtures enabled higher antioxidant levels in extracts compared to water–ethanol mixture and pure water due to its stronger hydrogen-bonding capacity, better solubility for polyphenols, greater stability under pressurized conditions, and enhanced mass transfer, making it more effective at preserving antioxidant capacity. Interestingly, in this research, we observed the same behavior for UAE, with its extracts exhibiting similar levels of ORAC antioxidant capacity compared to those from PLE (Figure 2c,d).

### 3.3. Polyphenol Profile of Olive Leaves

#### 3.3.1. Phenolic Acids

During the PLE of olive leaves, an increase from 50 to 70 °C combined with pure water, 15% water–ethanol, and 15% water–glycerol mixtures increased the recovery of gallic acid by 35%, 38%, and 52%, respectively (Table 1). Similarly, the extracts obtained by UAE were also favored with 30%, 19%, and 98% for 15% water–glycerol mixtures, increasing the recovery of gallic acid by 35%, 38%, and 52%, respectively (Table 1). A similar behavior was observed by Huaman et al. [29,32] where 50% water–glycerol mixtures at 150 °C recovered 18% more gallic acid content comparted to 50% water–ethanol mixtures under the same conditions. Regardless of the extraction method, glycerol can be more effective for extracting phenolic acids than ethanol due to its chemical structure, which contains three hydroxyl (-OH) groups. This characteristic enhances glycerol’s ability to form hydrogen bonds with phenolic acids, increasing their solubility and stability in the solvent [29]. The highest recovery of gallic acid was established with a 15%water–glycerol mixture at 70 °C both for PLE (5.37 µg/g dw) and UAE (5.94 µg/g dw) (Table 1). Olive leaves contain important concentrations of gallic acid compared to other acids like hydroxybenzoic acid, chlorogenic acid, caffeic acid, and vanillic acid [57,58].

#### 3.3.2. Stilbenes

The use of a 15% water–glycerol mixture at 70 °C in PLE resulted in the highest recovery of resveratrol (20.14 µg/g dry weight) compared to pure water (19.41 µg/g dry weight) and ethanol (17.64 µg/g dry weight) (Table 1). Similarly, the application of the 15% water–glycerol mixture during UAE also facilitated a higher recovery of resveratrol, yielding 19.15 µg/g dry weight (Table 1). Interestingly, previous studies using hot pressurized liquid extraction (HPLE) with 50% glycerol-water mixtures recovered 32% more resveratrol compared to 50% water–glycerol mixtures from Carmenère pomace [29,32]. In general, green extracts obtained from terrestrial leaves present a higher concentration of this compound. Rivera-Tovar et al. [31] reported the presence of 130 µg/g dw resveratrol in maqui leaves extracts obtained through the HPLE process, likely due to the effect of higher temperatures. In contrast, Guex et al. [58] identified the presence of this stilbene in olive leaves in minor amounts (0.048 µg/mL) under atmospheric conditions.

#### 3.3.3. Flavanols

Catechin, epicatechin, and procyanidin A2 were identified in PLE extracts observing a selective separation of them for higher temperatures (70 °C). Table 1 shows that the water–ethanol mixture was more effective in recovering catechin (10.20 µg/g dw) while the water–glycerol one recovered more epicatechin (8.94 µg/g dw) and procyanidin A2 (29.19 µg/g dw) content. Ethanol exhibits a dual nature as a polar and non-polar solvent, allowing it to interact favorably with functional groups of flavanols (phenolic and hydroxyl groups) [17].

On the contrary, the UAE combined with 15% water–glycerol mixture allowed the extraction of higher catechin (6.12 µg/g dw) and procyanidin (21.34 µg/g dw) concentrations (Table 1). Under atmospheric conditions, glycerol exhibits greater ability to donate hydrogen bonds (α: 1.21) compared to ethanol (α: 0.83) [50]; consequently, glycerol has greater capacity for solvation of these compounds.

#### 3.3.4. Flavonols

Contrary to the behavior of phenolic acids and stilbenes, flavonols were more soluble in water–ethanol mixtures for both extraction processes (PLE and UAE). In PLE, flavonols like kaempferol, quercetin, and rutin were more soluble in water–ethanol mixtures at 70 °C. Quercetin showed the highest recovery among these compounds using a 15% water–ethanol mixture at 70 °C (207.76 µg/g dw) (Table 2). Similarly, UAE enhanced the extractability of quercetin (118.24 µg/g dw) with 15% water–ethanol mixture at 70 °C (Table 2). Previous studies have reported that flavonols are more soluble with low ethanol concentrations (15%) [32]. Ethanol presents two fractions of polar (hydroxyl group) and non-polar (ethyl group) nature, which can interact with the aromatic and ketone-type carbonyl groups of flavonols, respectively [29].

#### 3.3.5. Phenylethanoids

During the PLE process, an increase in temperature from 50 to 70 °C combined with water–ethanol mixtures had a positive impact on the recovery of total phenylethanoids like hydroxytyrosol and tyrosol. The best conditions were established with 15% ethanol at 70 °C for hydroxytyrosol (9.44 µg/g dw) and tyrosol (17.79 µg/g dw) (Table 1). For the UAE process, when a 15% water–ethanol mixture at 70 °C was used, the recovery of hydroxytyrosol and tyrosol improved by 36% and 13% compared to 15% ethanol under the same conditions (Table 1). The ability of ethanol to dissolve hydroxytyrosol and tyrosol can be explained by the formation of hydrogen bonds between the hydroxyl groups of ethanol and phenylethanoids, as well as non-polar interactions between the aliphatic chain of the alcohol and the non-polar groups of hydroxytyrosol and tyrosol [59].

Contrary to the behavior of phenolic acids and stilbenes, flavonols were more soluble in water–ethanol mixtures for both processes (PLE and UAE). In the PLE, flavonols like kaempferol, quercetin, and rutin were more soluble in water–ethanol mixtures at 70 °C. Quercetin showed the highest recovery of these compounds using 15% ethanol at 70 °C (207.76 µg/g dw).

#### 3.3.6. Secoiridoid

Oleuropein is the most predominant polyphenol in olive leaves, and it has important bioactive properties [60]. In PLE, the use of 15% water–glycerol mixture at 70 °C improved the recovery of this compound by 71% and 17% compared to pure water and 15% water–ethanol mixture, respectively (Table 1). For the UAE process, the temperature increased from 50 to 70 °C improved the recovery of oleuropein by 1.74-, 1.71-, and 2.15-foldwith pure water, 15% ethanol, and 15% glycerol, respectively (Table 1). This positive effect of glycerol was reported by Sucharitha et al. [52] who indicated that the extraction of this compound through UAE was 1.34 times more effective when a 70% water–glycerol mixture was used as extraction solvent compared to the use of ethanol.

### 3.4. Principal Component Analysis of Extraction Process

**Figure 3:**
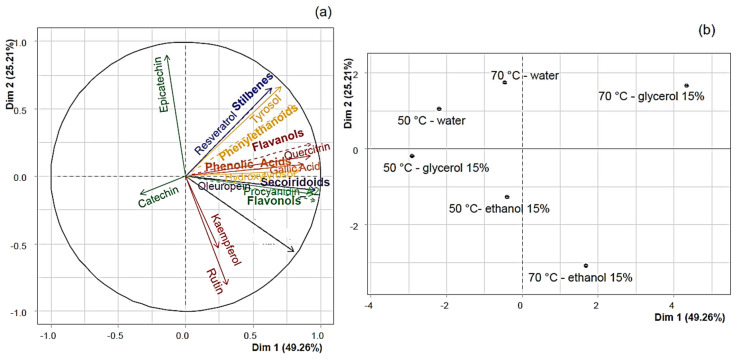

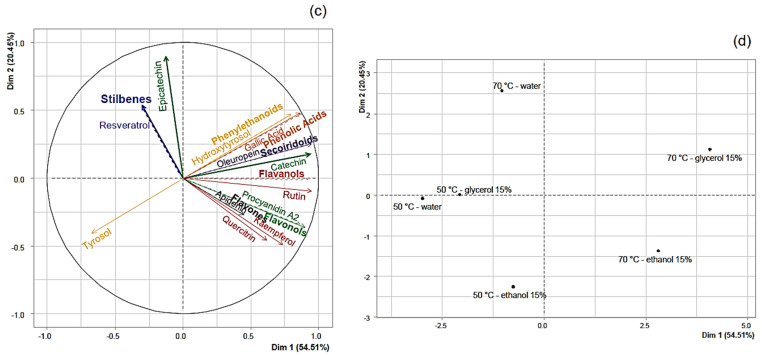
Principal Component Analysis of extraction process. PLE process (**a**,**b**); UAE process (**c**,**d**).

For the PLE process, Principal Component Analysis (PCA) presents the distribution of various phenolic compounds based on the first two principal components, which together account for 74.47% of the total variance (Figure 3a). Higher temperatures (70 °C) enhance the extraction of flavonols, which are located closer to the origin of the coordinate system (Figure 3b). Additionally, more polar solvents, such as water, preferentially extract polar compounds like flavonols and phenolic acids. In contrast, solvents such as ethanol and glycerol are more effective at extracting less polar compounds like stilbenes (Figure 3b).

For the UAE process, Principal Component Analysis (PCA) presents the distribution of specific phenolic compounds based on the first two principal components, which together account for 74.96% of the total variance (Figure 3c). Increasing the temperature to 70 °C enhances the solubility of these compounds in the solvent. Additionally, the polarity of the solvent plays an important role in the extraction of various families of polyphenols. For instance, stilbenes are more soluble in polar solvents like water, whereas secoiridoids and phenolic acids are better extracted using glycerol. In contrast, flavonols interact more effectively when ethanol is utilized (Figure 3d).

### 3.5. Modulation of Enzyme Activity

#### 3.5.1. α-Amylase Activity

**Figure 4:**
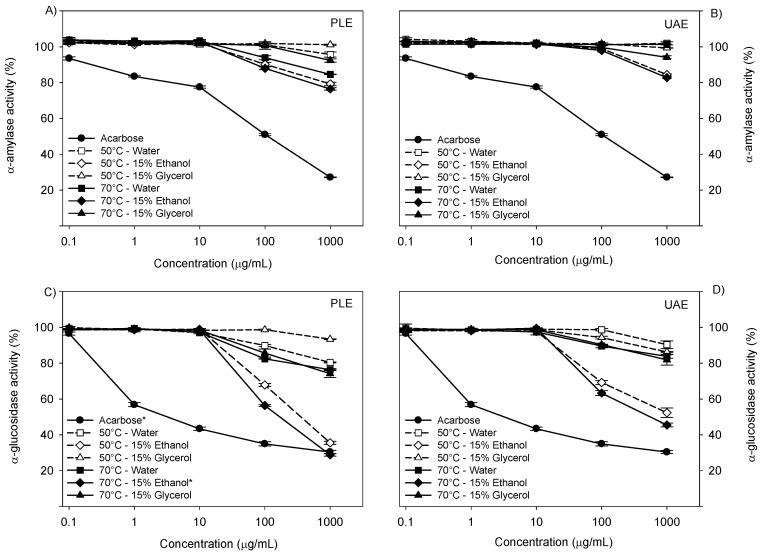
Effect of process conditions on the inhibition of enzymatic activity. Note: (**A**,**B**) represents the α-amylase activity for ELP and UAE, respectively; (**C**,**D**) represents the α-glucosidase activity for ELP and UAE, respectively. (*) indicates that there are no significant differences between the process conditions.

PLE and UAE extracts exhibited low α-amylase inhibitory activity as the temperature increased from 50 to 70 °C (Figure 4). For PLE at 70 °C, the inhibition values were 15% for the pure water extract, 8% for the 15% water–ethanol extract, and 24% for the 15% water–glycerol extract at 1000 µg/mL (Figure 4A). UAE extracts obtained with pure water at 70 °C and 1000 µg/mL showed no inhibition of the enzyme. Additionally, the water–ethanol and water–glycerol mixtures exhibited inhibitions of 17% and 6% in α-amylase activity, respectively (Figure 4B). In contrast, acarbose demonstrated a significantly higher inhibition of 73% at the same concentration.

In general, polyphenols should inhibit α-amylase through interactions between their hydroxyl groups and the catalytic site of the enzyme, as well as through hydrophobic forces between the aromatic rings of polyphenols and the tryptophan residues of α-amylase [61]. Some studies have reported the low effectiveness of polyphenolic extracts to inhibit this enzyme, which could be beneficial for glycemic homeostasis [62,63]. While inhibiting both amylase and glucosidase is a therapeutic goal in diabetes management, non-specific inhibition of these enzymes should be avoided, as it may lead to adverse side effects and disrupt normal digestion. For instance, acarbose, a non-specific inhibitor, can interfere with other gastrointestinal functions, causing discomfort, flatulence, and diarrhea. In contrast, polyphenolic extracts act as specific inhibitors of these enzymes, effectively regulating blood glucose levels without compromising digestion [39,61].

#### 3.5.2. α-Glucosidase Activity

The PLE extracts obtained at 70 °C using 15% water–ethanol and 15% water–glycerol mixtures and pure water reduced α-glucosidase activity by 71%, 25%, and 23%, respectively (Figure 4C). Notably, the extracts with the 15% water–ethanol mixture at 70 °C demonstrated effectiveness similar to acarbose in inhibiting this enzyme under the same conditions (Figure 4C). Meanwhile, UAE extracts combined with the 15% water–ethanol mixture at 70 °C reduced enzymatic activity by 45% (Figure 4D). Some studies have shown that certain plants can inhibit α-glucosidase. Alshaal et al. [64] reported that the extract obtained with 70% ethanol at 75 °C inhibits the α-glucosidase activity of 81.34% with olive leaves under the UAE process. Mansour et al. [65] reported that using pure water at 45 °C reduces the α-glucosidase activity by 92.46% with olive leaves under the conventional process. The α-glucosidase enzyme is responsible for breaking down oligosaccharides and disaccharides into glucose [66]. Thus, inhibitors of this enzyme can slow down this process and potentially help in treating type 2 diabetes mellitus. This ability of olive leaf extracts is probably due to the presence of oleuropein and tyrosol, two compounds that are hydroxylated in Positions 5, 6, and 7 compared to gallic acid and catechin, which are hydroxylated in Positions 3, 4, and 5 and Positions 3,5 and 7, respectively. This particular characteristic allows the extracts to interact with the enzyme’s active sites, reducing its ability to hydrolyze polysaccharides [39].

## 4. Conclusions

For both PLE and UAE, the use of water–glycerol mixtures at 70 °C was more effective in obtaining extracts rich in polyphenols, which exhibited strong antioxidant properties. The DPPH assay demonstrated lower IC_50_ values, indicating enhanced antioxidant capacity, further supported by higher ORAC values. The 15% water–glycerol mixture was particularly efficient in extracting phenolic acids, stilbenes, and secoiridoids, whereas the 15% water–ethanol mixture was more effective in recovering flavonols and phenylethanoids. Additionally, the extracts exhibited significant α-glucosidase inhibitory activity comparable to acarbose, a standard antidiabetic drug. Finally, these findings highlight the potential for developing functional foods or supplements derived from agro-industrial waste to aid in the management of diabetes. However, further exploration of other agro-industrial wastes as sources of phenolic compounds, along with the evaluation of the long-term effects of these extracts on diabetes management in clinical trials, is needed.

## Figures and Tables

**Table 1:** Analytical parameters for the quantification of specific polyphenols.

Specific Polyphenol	Wavelength (nm)	Regression Equation	R^2^
Oleuropein	254	Y = 28.1342X − 0.3345	0.9997
Tyrosol	260	Y = 67.5463X − 1.0865	0.9999
Hydroxytirosol	260	Y = 59.2803X − 0.7963	0.9999
Gallic	270	Y = 6.615X + 1.7801	0.9992
Quercitin	270	Y = 24.4618X + 2.282	0.9997
Procyanidin A2	270	Y = 149.1198X + 0.9753	0.9999
Catechin	280	Y = 25.2511X − 0.5309	0.9999
Epicatechin	280	Y = 43.3950X − 2.1554	0.9998
Rutin	280	Y = 141.9918X − 5.4568	0.9998
Resveratrol	324	Y = 78.8100X − 3.3578	0.9997
Kaempferol	373	Y = 38.0226X − 1.5363	0.9997

**Table 2:** Polyphenol profile of the extracts obtained.

Description		ELP Process				UAE Process		
		Pure Water	15% Ethanol	15% Glycerol		Pure Water	15% Ethanol	15% Glycerol
		Mean ± DE	Mean ± DE	Mean ± DE		Mean ± DE	Mean ± DE	Mean ± DE
**Phenolic Acids** (µg/g dw)								
Gallic acid	50 °C	3.59 ± 0.17	3.65 ± 0.15	3.53 ± 0.09		3.00 ± 0.15	3.08 ± 0.18	2.70 ± 0.21
	70 °C	4.85 ± 0.23	5.04 ± 0.11	5.37 ± 0.52		3.94 ± 0.39	3.58 ± 0.15	5.94 ± 0.43
**Stilbenes** (µg/g dw)								
Resveratrol	50 °C	18.26 ± 0.09	18.11 ± 0.08	17.04 ± 0.17		17.21 ± 0.67	17.70 ± 0.93	17.19 ± 0.83
	70 °C	19.41 ± 0.27	17.64 ± 0.38	20.14 ± 0.24		17.69 ± 0.16	18.10 ± 0.21	19.15 ± 0.29
**Flavanols** (µg/g dw)								
Catechin	50 °C	8.60 ± 0.51	3.39 ± 0.17	7.99 ± 0.10		5.92 ± 0.29	4.34 ± 0.35	6.90 ± 0.32
	70 °C	9.71 ± 0.38	10.20 ± 0.86	4.24 ± 0.38		5.41 ± 0.42	4.13 ± 0.12	6.12 ± 0.45
Epicatechin	50 °C	9.14 ± 0.65	3.09 ± 0.15	8.18 ± 0.60		4.64 ± 0.28	3.56 ± 0.35	7.49 ± 0.35
	70 °C	8.38 ± 0.56	2.96 ± 0.24	8.94 ± 0.88		8.68 ± 0.78	3.09 ± 0.21	7.00 ± 0.67
Procyanidin A2	50 °C	12.95 ± 0.63	21.94 ± 2.06	10.99 ± 0.64		6.71 ± 0.44	15.50 ± 0.92	12.04 ± 0.81
	70 °C	15.23 ± 1.20	22.40 ± 0.89	29.19 ± 2.05		7.47 ± 0.13	19.77 ± 0.83	21.34 ± 1.22
**Flavonols** (µg/g dw)								
Kaempferol	50 °C	2.15 ± 0.06	2.93 ± 0.08	2.26 ± 0.07		1.90 ± 0.06	2.16 ± 0.07	2.06 ± 0.04
	70 °C	2.27 ± 0.15	2.48 ± 0.13	2.39 ± 0.14		1.89 ± 0.04	2.82 ± 0.17	2.27 ± 0.10
Quercetin	50 °C	64.86 ± 5.78	108.10 ± 2.28	81.23 ± 1.45		28.58 ± 1.59	100.21 ± 0.87	88.86 ± 1.29
	70 °C	87.60 ± 1.93	207.76 ± 2.29	113.76 ± 1.40		18.96 ± 0.44	118.24 ± 2.17	82.53 ± 0.85
Rutin	50 °C	ND	1.68 ± 0.16	ND		ND	3.29 ± 0.16	ND
	70 °C	ND	53.08 ± 2.38	ND		1.45 ± 0.11	63.26 ± 0.37	57.43 ± 1.04
**Phenylethanoids** (µg/g dw)								
Hidroxytyrosol	50 °C	7.70 ± 0.33	6.39 ± 0.37	5.87 ± 0.80		4.89 ± 0.44	6.40 ± 0.57	6.70 ± 0.24
	70 °C	6.87 ± 0.46	8.63 ± 0.14	9.44 ± 0.43		28.78 ± 1.92	30.05 ± 0.19	41.95 ± 0.94
Tyrosol	50 °C	12.76 ± 0.67	13.40 ± 0.12	10.41 ± 0.58		4.72 ± 0.25	2.81 ± 0.19	6.49 ± 0.16
	70 °C	17.21 ± 0.29	17.79 ± 0.66	11.92 ± 0.47		12.28 ± 0.14	13.49 ± 0.26	11.84 ± 0.19
**Secoiridoid** (µg/g dw)								
Oleuropein	50 °C	78.19 ± 2.69	93.63 ± 0.64	87.49 ± 1.00		104.82 ± 4.02	132.80 ± 5.66	115.00 ± 1.39
	70 °C	100.19 ± 1.87	146.87 ± 2.85	171.48 ± 3.72		182.79 ± 3.38	226.85 ± 2.44	246.70 ± 2.41

## Data Availability

The original contributions presented in the study are included in the article, further inquiries can be directed to the corresponding author.
